# *Halomonas elongata*: a microbial source of highly stable enzymes for applied biotechnology

**DOI:** 10.1007/s00253-023-12510-7

**Published:** 2023-04-13

**Authors:** Ana I. Benítez-Mateos, Francesca Paradisi

**Affiliations:** Department of Chemistry, Biochemistry and Pharmaceutical Sciences, University of Bern, Bern, Switzerland

**Keywords:** Halomonas, Halotolerant, Enzyme, Co-solvent stability, Biocatalysis, Biotechnology

## Abstract

**Abstract:**

Extremophilic microorganisms, which are resistant to extreme levels of temperature, salinity, pH, etc., have become popular tools for biotechnological applications. Due to their availability and cost-efficacy, enzymes from extremophiles are getting the attention of researchers and industries in the field of biocatalysis to catalyze diverse chemical reactions in a selective and sustainable manner. In this mini-review, we discuss the advantages of *Halomonas elongata* as moderate halophilic bacteria to provide suitable enzymes for biotechnology. While enzymes from *H. elongata* are more resistant to the presence of salt compared to their mesophilic counterparts, they are also easier to produce in heterologous hosts compared with more extremophilic microorganisms. Herein, a set of different enzymes (hydrolases, transferases, and oxidoreductases) from *H. elongata* are showcased, highlighting their interesting properties as more efficient and sustainable biocatalysts. With this, we aim to improve the visibility of halotolerant enzymes and their uncommon properties to integrate biocatalysis in industrial set-ups.

**Keypoints:**

*• Production and use of halotolerant enzymes can be easier than strong halophilic ones.*

*• Enzymes from halotolerant organisms are robust catalysts under harsh conditions.*

*• Halomonas elongata has shown a broad enzyme toolbox with biotechnology applications.*

## Introduction

Industrial processes are expanding towards the synthesis of a broader variety of molecules. Hence, catalysts are required more and more to tolerate a range of conditions that may include harsh reaction environments such as the presence of organic co-solvents, extreme pH, extreme temperature, etc., which are often employed at industrial set-ups. Enzymes from different organisms can be applied as very selective biocatalysts in synthetic procedures (Katsimpouras and Stephanopoulos 2021; Winkler et al. 2021). Indeed, the global enzyme market is growing steadily with a projected value of $8.7 billion in 2026 (Research B 2021). However, enzymes present a limited stability under typical industrial conditions due to their natural origin, hampering the progress of industrial biotechnology. Extremozymes are the exception; these unique proteins are produced by organisms adapted to live in extreme environments (i.e., halophiles, thermophiles, basophiles); thus, since their discovery, there is a growing interest in their potential and biotechnological application (Littlechild 2015; Sani and Rathinam 2018; Mesbah 2022). Thermophiles are by far the most well-known type of extremophilic organisms. Thermophiles are often found in volcanic zones and hot springs, and they have been studied for decades and had a pivotal role in important breakthroughs as the use of the *Taq* polymerase for PCR (polymerase chain reaction) tests (Saik et al. 1987). At the other end, among the most recently discovered extremophiles are the barophiles (also known as piezophiles), which are often found in the deepest sea bottom under high levels of pressure (Fang et al. 2010). Yet, their mechanisms of adaptation are not completely deciphered.

Unlike thermophiles and, to some extent, also basophiles, halophilic microorganisms are yet unexploited in biotechnology despite their distinguished features (Yin et al. 2015; Daoud and Ben Ali 2020). According to their salt requirement for growth, halophiles are classified into three main categories: extreme halophiles (15–25% NaCl), moderate halophiles (3–15% NaCl), and mild halophiles (1–3% NaCl) (Delgado-García et al. 2012). However, some moderate and mild halophiles can tolerate higher salt concentrations even though this is not a requirement for their growth. This type of microorganisms is termed as halotolerant microorganism. Enzymes from halotolerant organisms are indeed an interesting biological toolbox as they are stable under a wider range of conditions, including low salt environments, which is normally not an option for enzymes from true halophilic species. In addition to halotolerance, many halophilic organisms can tolerate high pH and a broad temperature range, acting as “polyextremophiles” (Yin et al. 2015). Hence, halophilic organisms can be utilized under conditions in which their mesophilic counterparts cannot survive.

In 1980, a new moderate halophile bacterium named *Halomonas elongata* was isolated from a solar salt facility in Bonaire, Netherlands Antilles (Vreeland et al. 1980). Since then, the aerobic and gram-negative γ-proteobacteria have found various biotechnological applications such as host strain for heterologous protein expression (Frillingos et al. 2000), whole-cell biocatalyst (Tanimura et al. 2013; Chen et al. 2022; Dutta and Bandopadhyay 2022), and biosynthesis of nanoparticles (Taran et al. 2018). Yet, the most popular use of *H. elongata* relies on the production of ectoine (Tanimura et al. 2013; Ng et al. 2023). This osmolyte is accumulated inside the cells of *H. elongata* as an adaptation mechanism to provide an osmotic equilibrium with respect to the hyperosmotic environment. Ectoine is used as a cell protectant and as enzyme stabilizer, and it is produced annually at multi-ton industrial scale because of its health care applications (Pastor et al. 2010). Noteworthily, *H. elongata* is classified as a moderate halophile and halotolerant, as it does not require salt for growth but can tolerate medium to high salt concentrations (5–25% NaCl). This is indeed an advantage, as the cytoplasmatic environment of *H. elongata* does not present hypersalinity and its enzymes can be produced, correctly folded, by heterologous expression in conventional hosts such as the mesophilic *Escherichia coli*, (Cerioli et al. 2015) avoiding less-common expression systems, which are required for extreme halophilic proteins. Consequently, *H. elongata* is an ideal candidate to provide stable enzymes that could be used in industrial processes.

**Fig. 1:**
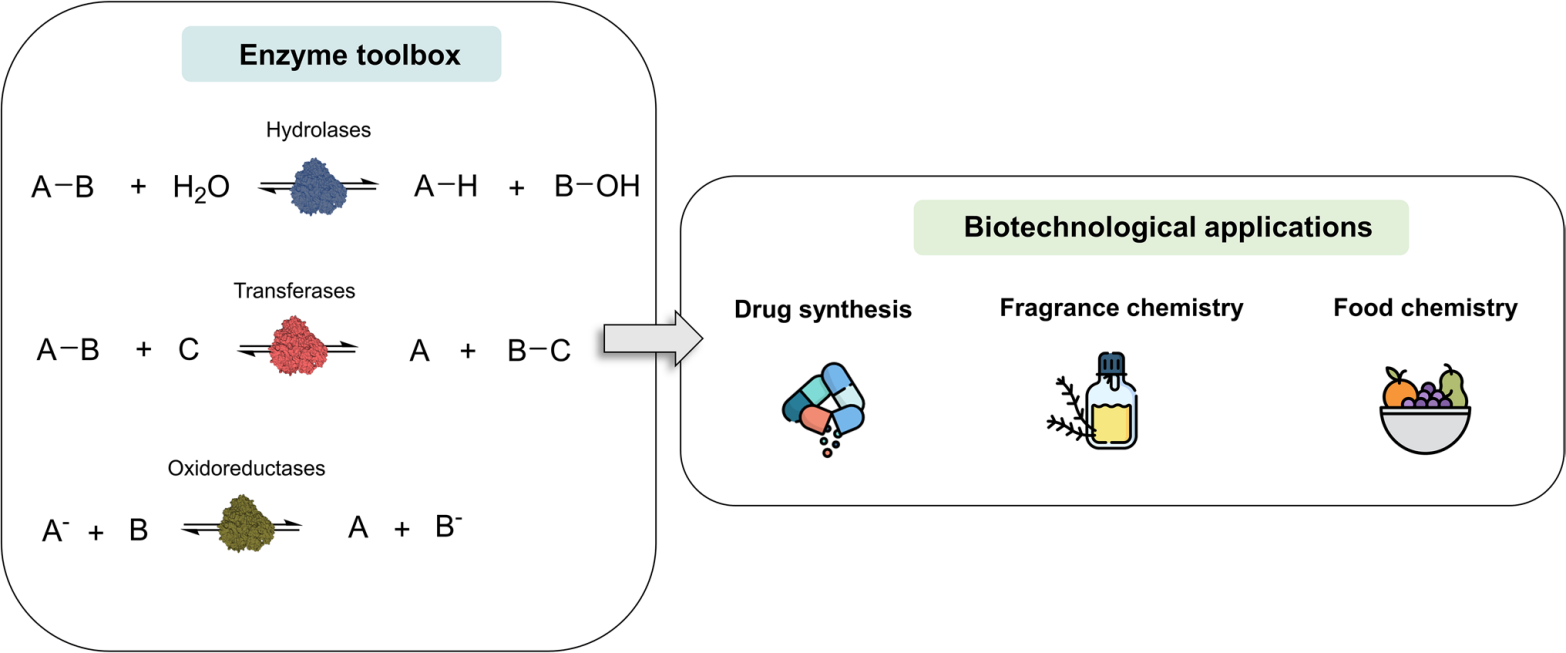
Overview of the application of enzymes from *H. elongata.* Reaction schemes represent the main types of enzymatic reactions described by using enzymes from *H. elongata*

Clearly, recent reviews covering biotechnological applications of extremophiles are strongly focused on thermophilic enzymes, which have been already broadly exploited (Littlechild 2015; Mesbah 2022). On the other hand, specific reviews on halophilic enzymes are dedicated to extreme halophiles (DasSarma and DasSarma 2015; Daoud and Ben Ali 2020) whose properties differ from moderate halophilic microorganisms such as *H. elongata.* Herein, the potential of halotolerant enzymes in biotechnological processes is highlighted, with a particular focus on the tolerance to organic co-solvents. Specifically, we would like to encourage biotechnology researchers to explore the broad toolkit of enzymes from *Halomonas elongata* as robust biocatalysts with potential applications in bioenergy, bioremediation, biomedicine, food industry, and pharmaceutical synthesis, among others (Fig. 1).

## Salt-tolerance mechanism and benefits for biotechnology

Two alternative strategies for salt-tolerance are adopted by different microorganisms. The simplest mechanism consists of the accumulation of molar concentrations of KCl in the cytoplasm to provide osmotic balance to the cells. Halophilic archaea as a few bacteria follow this strategy to survive in high-salt environments (Kelefiotis-Stratidakis et al. 2019). Alternatively, the “compatible solutes” method is based on the accumulation of organic osmotic solutes such as glycine, ectoine, glycerol, simple sugars, etc. These molecules can be rapidly produced in bacteria according to the outside salt concentration (Galinski 1995). Many bacteria including *Halomonas elongata* use this strategy to adapt to hypersaline environments. Interestingly, some species such as *Halorhodospira halophila* can use both strategies, thus adapting to the environment without the obligation of salt-in halophiles (Oren 2013).

**Fig. 2:**
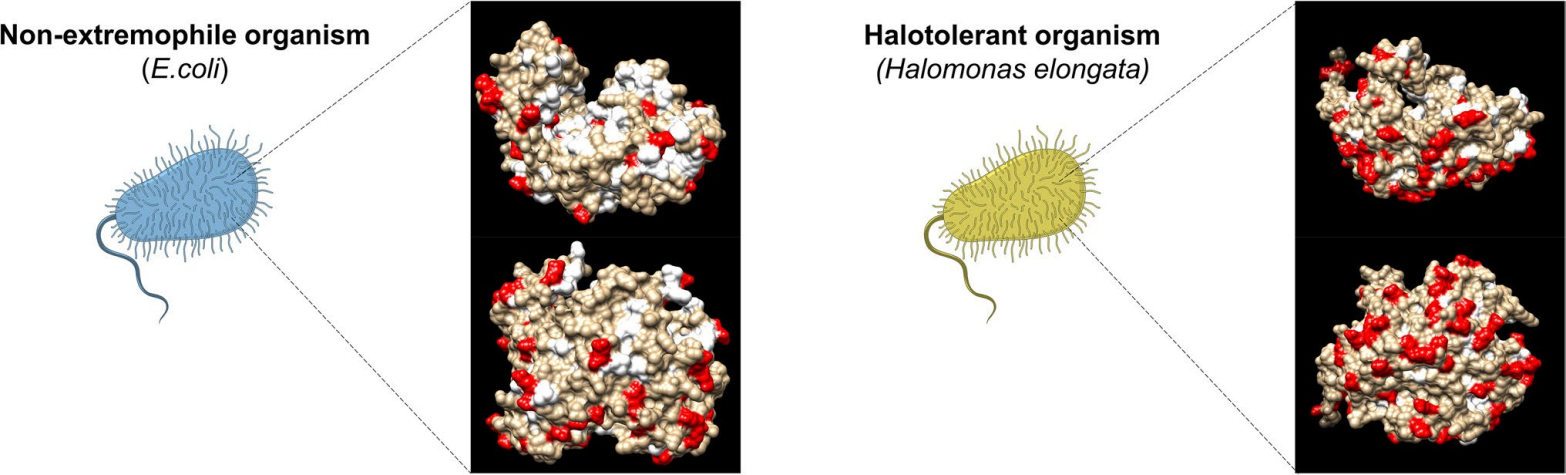
Comparison of the residue composition of a halotolerant aminotransferase enzyme (Uniprot: E1V913) and its homolgous from *E. coli* (Uniprot: P42588). Asp and Glu residues are highlighted in red, while Phe, Leu, and Ile are depicted in white

Halophilic and halotolerant enzymes have proved to be more resistant to high-salt concentrations even when expressed in mesophilic hosts or after protein purification when the adaptative mechanism described above cannot take place. What makes them special? In the 1990s, when the first structure of a halophilic enzyme was solved, a more detailed perspective at the molecular level started to develop. The analysis of the amino acid content of extreme halophilic enzymes revealed a high density of negatively charged residues (aspartic and glutamic acid) on the protein surface (DasSarma and DasSarma 2015) (Fig. 2). Moreover, genome sequencing and bioinformatic studies showed a lowprotein isoelectric point between 4 and 5, while non-halophilic organisms (i.e., *E. coli*) present a more homogeneous distribution of acidic and basic proteins with an isoelectric point near to neutrality (Kennedy et al. 2001). Indeed, it has been shown that negatively charged residues are a key requirement for maintaining the solubility and activity of enzymes under water-limited conditions. In fact, hypersalinity offers a diminished overall amount of water to the protein as the water molecules are displaced by the salt ions. The formation of hydrogen bonds between side chains of the negatively charged residues and water molecules is crucial to maintain the hydration shell in low-water conditions (DasSarma and DasSarma 2015). Additionally, a correlation has been found between the degree of negatively charged residues and the salt dependency, as extreme halophilic proteins present high negativity compared to halotolerant proteins (DasSarma and DasSarma 2015).

Apart from their negatively charged exposed residues, the composition of bulky hydrophobic residues (leucine, isoleucine, and phenylalanine) on the surface of halophilic enzymes is poorer than in mesophiles (Fig. 2). Instead, halophilic proteins contain smaller and/or less hydrophobic residues (alanine, glycine, serine, and threonine) (Kastritis et al. 2007). By reducing the hydrophobic patches on the protein surface, the surface hydration is also enhanced. Furthermore, these correlations agree with increased flexibility of halophilic proteins in low-water conditions. Noteworthily, this peculiar pattern is mainly attributed to extreme halophilic enzymes, which are normally exposed to a hypersaline cytoplasmatic environment. In the case of moderate or mild halophiles such as *H. elongata*, not all enzymes follow this pattern.

Dehydration could be compared with aqueous solutions containing organic solvents, where the ratio of water molecules to the solute (or the protein) is significantly decreased. Therefore, halophilic and halotolerant enzymes can maintain significant activity and stability in presence of organic solvents. Such a tolerance is highly desirable to implement biocatalysts at industrial processes when the presence of a co-solvent is needed because of the poor water solubility of many substrates (Yin et al. 2015). A remarkable case of tolerance to organic solvents was displayed by a purine nucleoside phosphorylase from *H. elongata.* The enzyme retained > 60% activity after 72 h in presence of 50% DMSO (Benítez-Mateos and Paradisi 2022).

High-salt tolerance is also an attractive feature from an industrial perspective to reduce the process costs and improve the efficiency. Many biotechnological processes are not feasible at industrial scale due to the excessive demand of fresh water, the energy required for sterilization of bioreactors, and the discontinuity of bioreactions caused by biological contamination. The application of halotolerant enzymes can alleviate all those problems, as higher salt concentrations can be used during biotransformations, thus preventing contaminations, and reducing costs associated with sterilization and high demands of water (Mokashe et al. 2018). In addition, many (moderate) halophilic enzymes, in particular the genre *Halomonas*, can tolerate a more basic pH, avoiding biological contamination as well.

## Hydrolases

Hydrolases are a group of enzymes (including proteases, lipases, esterases, amylases, etc.) that catalyze the cleavage of molecular bonds in presence of water. On the other hand, hydrolases can also catalyze synthetic reactions in presence of organic co-solvents where the thermodynamic equilibrium is shifted due to the low water activity. Biosynthetic reactions catalyzed by hydrolases are indeed highly attractive in industry, being almost 75% of all industrial enzymes (Busto et al. 2010). However, the use of mesophilic hydrolases in organic co-solvents may pose a challenge as their activity and stability can decrease dramatically under such harsh conditions. Extensive effort has been made to improve the properties of mesophilic enzymes, for instance genetic modifications and enzyme immobilization, which increase costs and time for the biocatalyst preparation. In contrast, halotolerant enzymes are naturally adapted to function under water-limited conditions (Mesbah 2022). Therefore, halotolerant hydrolases could be a suitable and efficient alternative for biosynthetic processes in industry (Delgado-García et al. 2012).

To date, a few hydrolases from *H. elongata* with potential applications in pharmaceutical, biofuel, and food industry as well as biomedicine have been described (Table 1). One of those esterases is HeE, which has been used for the hydrolysis of non-steroidal anti-inflammatory drugs (NSAIDS) in presence of 10% of organic co-solvent (Roura Padrosa et al. 2019). In another example, a new esterase belonging to the YbfF family presented a maximum activity at high salt exposure such as 0.5–4 M NaCl (Yoo et al. 2020)*.* The tolerance to medium–high salt concentration, while not a requirement, is a key advantage for biomedical applications as shown for the L-asparaginase from *H. elongata* (Ghasemi et al. 2017)*.* L-asparaginases are interesting therapeutics for anticancer treatment; however, mesophilic enzymes have exhibited up to 80% loss of activity in 0.9% (~ 0.16 M) saline solution. As a consequence of its halotolerant origin, L-asparaginase from *H. elongata* was easily expressed in *E. coli*, purified, and successfully applied in antitumor tests, presenting comparable anticancer potential to commercial asparaginases while maintaining the activity in saline serum (Ghasemi et al. 2017). Remarkably, no cytotoxicity was observed even at very high doses.

**Table 1:** List of enzymes from *Halomonas elongata* with (potential) biotechnological applications. Enzyme production was carried out by heterologous expression in *E. coli*

Entry	Enzyme	Biotechnological application	Reference
1	L-Asparaginase	Chemotherapeutic agent	(Ghasemi et al. 2017)
2	Esterase	Synthesis of anti-inflammatory drugs	(Roura Padrosa et al. 2019)
3	YbfF esterase	Not described yet	(Yoo et al. 2020)
4	DABA transaminase	Pharmaceutical synthesis Cosmetic industry	(Ono et al. 1999; Wang et al. 2021)
5	DABA acetyltransferase		
6	Ectoine synthase		
7	ω-Transaminase	Pharmaceutical synthesis Food chemistry Polymer synthesis	(Cerioli et al. 2015; Planchestainer et al. 2017; Roura Padrosa et al. 2020)
8	Purine nucleoside phosphorylase	Synthesis of anticancer and antiviral drugs	(Benítez-Mateos and Paradisi 2022)
9	Thymidine phosphorylase	Synthesis of anticancer and antiviral drugs	(Benítez-Mateos et al. 2022)
10	Alcohol dehydrogenase	Flavor chemistry	(Contente et al. 2020)
11	Alanine dehydrogenase	Flavor and fragrance chemistry	(Roura Padrosa et al. 2021)
12	Choline dehydrogenase	Not described yet	(Gadda and McAllister-Wilkins 2003)
13	Azoreductase	Waste-water treatment	(Eslami et al. 2016)
14	Imine reductase (pyrroline-5-carboxylate reductase)	Pharmaceutical synthesis	(Roura Padrosa et al. 2020)

## Transferases

The transferring of a specific functional group (i.e., amino, carbonyl, methyl, carboxyl, acetyl, phosphate) from a *donor molecule* to another *acceptor molecule* is catalyzed by transferases. Three of the most well-known transferases in *H. elongata* are DABA transaminase, DABA acetyltransferase, and ectoine synthase, which are part of the ectoine synthesis route (Ono et al. 1999). Ectoine is a highly interesting natural product used as bioactive ingredient in pharmaceutics and cosmetics. Because of the great salt adaptability of *H. elongata*, the bacterial cells can be cultured in high salinity conditions to promote the ectoine synthesis followed by low salinity conditions to release the ectoine that is naturally accumulated as an osmolyte (Sauer and Galinski 1998). Moreover, the synthetic process was performed in an open unsterile medium, facilitating operational aspects, and reducing costs. Notwithstanding, ectoine production is not limited to *H. elongata* cells. The halotolerant enzymes can be produced by heterologous expression in other microorganisms offering an easy-to-handle strategy since more sophisticated fermentation technologies are avoided (Wang et al. 2021).

Another example of a transferase from *H. elongata* with many potential biotechnological applications is the *S*-selective ω-transaminase, also known as HewT. This enzyme showed a broad substrate scope and a high tolerance to a variety of organic co-solvents (e.g., methanol, ethanol, DMSO, isopropanol) up to 20% (Cerioli et al. 2015). Harnessing these properties that other homologous transaminases do not possess, HewT has been extensively exploited for the synthesis of high-value molecules in pharmaceutical synthesis (Contente and Paradisi 2018; Cairns et al. 2019; Contente et al. 2019; Hegarty and Paradisi 2020; Roura Padrosa et al. 2020; Heckmann et al. 2021; Romero-Fernandez and Paradisi 2021), polymer chemistry (Romero-Fernandez et al. 2022), food chemistry (Planchestainer et al. 2017; Benítez-Mateos et al. 2018, 2021; Roura Padrosa et al. 2021), and other applications (Contente et al. 2016; Planchestainer et al. 2019; Czarnievicz et al. 2022).

In the last years, nucleoside phosphorylases are getting increasing attention for their biocatalytic potential on the biosynthesis of antiviral and anticancer drugs such as Islatravir (Huffman et al. 2019). The main challenge of these reactions is the poor water solubility of the substrates (nucleobases and sugar donors). Consequently, high co-solvent concentrations are needed, causing the inactivation of the enzymes. Once again, *H. elongata* has proved to be a valuable source of robust enzymes. A purine nucleoside phosphorylase (HePNP) and a thymidine phosphorylase (HeTP) have been recently characterized showing an astonishing stability in presence of 50% DMSO (50% activity after 3 days) and 10% ethanol (80% after 3 h), respectively (Benítez-Mateos et al. 2022; Benítez-Mateos and Paradisi 2022). The great operational stability of both enzymes upon immobilization on porous particles enabled the synthesis of valuable pharmaceutical molecules reaching the maximum yields reported so far for those enzymatic reactions.

## Oxidoreductases

Enzymes that catalyze the electron transfer from one molecule, the reductant, to another molecule, the oxidant, are denominated oxidoreductases. Although these enzymes are very interesting for oxidation reactions in a more sustainable manner, industry has not embraced completely oxidoreductases due to their incompatibility with high substrate concentrations, strongly oxidative conditions, and cofactor dependency, which is economically disadvantageous (Martínez et al. 2017).

A remarkable alcohol dehydrogenase from *H. elongata* is the so-called HeADH-II. This enzyme showed an uncommon overoxidative catalytic activity, enabling direct overoxidation of primary alcohols to carboxylic acids, which is normally achieved by multi-enzyme systems that are less atractive for industry as different enzymes may require different reaction conditions (Contente et al. 2020). In addition, HeADH-II presented a high stability in the presence of water-miscible organic co-solvents, proving its applicability in the synthesis of food additives. Another enzyme with potential for food chemistry is alanine dehydrogenase, HeAlaDH, which showed an excellent stability in presence of co-solvents up to 20% as well as high tolerance (> 70% after 48 h) to pH ranging from 5 to 10 (Roura Padrosa et al. 2021; Marchini et al. 2022).

Regarding applications for environmental biotechnology, a choline dehydrogenase and an azoreductase from *H. elongata* exhibited outstanding properties. Choline dehydrogenases are of considerable interest to enhance different stress tolerances (hypersalinity, freezing, high temperatures, etc.) of transgenic plants, as these enzymes catalyze the oxidation of choline to glycine-betaine that is used as an osmolyte in cells. The enzyme from *H. elongata* is a very suitable candidate as it can be easily expressed and presents a great stability compared with its homologous from *E. coli*, *Pseudomonas* or rat liver mitochondria (Gadda and McAllister-Wilkins 2003). In the case of azoreductase from *H. elongata*, water treatment to eliminate azo dyes produced in textile, paper, or food industries was the targeted process. Waste effluents contain many salts that hamper bioremediation processes with mesophilic microorganisms. In contrast, the azoreductase from *H. elongata* retained more than 50% of activity in presence of NaCl concentration as high as 50 g/L (Nakhaee et al. 2018). Furthermore, the thermostability of the enzyme was enhanced by introducing a simple disulfide bond.

## Conclusion and future perspectives

Even though enzymes from diverse halotolerant bacteria have been described, *Halomonas elongata* has attracted much attention due to the broad range of salinity that it can tolerate without depending directly on salt for growth, facilitating the heterologous expression of halotolerant proteins in common expression system such as *E. coli*. Moreover, the flexibility of *H. elongata* can be also appreciated on the wide range of temperature and pH that its enzymes can often tolerate. The reason for such a high stability seems to rely on the amino acid composition on the protein surface. Then, it may be plausible to think that a mesophilic enzyme could be “transformed” into a more halophilic enzyme by increasing the number of negatively charged and hydrophilic residues. This was, indeed, questioned by Qvist et al., but 6 residue changes from lysine to aspartate were not enough to observe any significant stabilizing effect (Qvist et al. 2012).

Undoubtedly, different enzymes from *H. elongata* have shown an unusual stability that prolongs the lifespan of the biocatalyst and broadens the possibilities for which to use enzyme catalysis (Table 1). On another note, the high density of negative charges on the protein surface could be an advantage for enzyme immobilization on supports activated with positive charges (i.e., amino groups). Therefore, harnessing the unique properties of the halotolerant enzyme toolbox can overcome different challenges on the path toward more efficient and sustainable chemistry.

## References

[CR1] Benítez-Mateos AI, Bertella S, Behaghel de Bueren J, Luterbacher J, Paradisi F (2021). Dual revalorization of lignin through its use as a versatile and renewable matrix for enzyme immobilization and (flow)bioprocess engineering. Chemsuschem.

[CR2] Benítez-Mateos AI, Contente ML, Velasco-Lozano S, Paradisi F, López-Gallego F (2018) Self-sufficient flow-biocatalysis by coimmobilization of pyridoxal 5′-phosphate and ω-rransaminases onto porous carriers. ACS Sustain Chem Eng 6 10.1021/acssuschemeng.8b02672

[CR3] Benítez-Mateos AI, Klein C, Roura Padrosa D, Paradisi F (2022) A novel thymidine phosphorylase to synthesize (halogenated) anticancer and antiviral nucleoside drugs in continuous flow. Catal Sci Technol 6231–6238 10.1039/d2cy00751g 10.1039/d2cy00751g PMC9575728 36325519

[CR4] Benítez-Mateos AI, Paradisi F (2022). Sustainable Flow-Synthesis of (Bulky) Nucleoside drugs by a novel and highly stable nucleoside phosphorylase immobilized on reusable supports. Chemsuschem.

[CR5] Busto E, Gotor-Fernández V, Gotor V (2010). Hydrolases: catalytically promiscuous enzymes for non-conventional reactions in organic synthesis. Chem Soc Rev.

[CR6] Cairns R, Gomm A, Ryan J, Clarke T, Kulcinskaja E, Butler K, O’Reilly E (2019). Conversion of aldoses to valuable ω-amino alcohols using amine transaminase biocatalysts. ACS Catal.

[CR7] Cerioli L, Planchestainer M, Cassidy J, Tessaro D, Paradisi F (2015). Characterization of a novel amine transaminase from *Halomonas elongata*. J Mol Catal B Enzym.

[CR8] Chen GQ, Zhang X, Liu X, Huang W, Xie Z, Han J, Xu T, Mitra R, Zhou C, Zhang J, Chen T (2022). *Halomonas spp*., as chassis for low-cost production of chemicals. Appl Microbiol Biotechnol.

[CR9] Contente ML, Farris S, Tamborini L, Molinari F, Paradisi F (2019). Flow-based enzymatic synthesis of melatonin and other high value tryptamine derivatives: a five-minute intensified process. Green Chem.

[CR10] Contente ML, Fiore N, Cannazza P, RouraPadrosa D, Molinari F, Gourlay L, Paradisi F (2020). Uncommon overoxidative catalytic activity in a new halo-tolerant alcohol dehydrogenase. ChemCatChem.

[CR11] Contente ML, Paradisi F (2018). Self-sustaining closed-loop multienzyme-mediated conversion of amines into alcohols in continuous reactions. Nat Catal.

[CR12] Contente ML, Planchestainer M, Molinari F, Paradisi F (2016). Stereoelectronic effects in the reaction of aromatic substrates catalysed by: *Halomonas elongata* transaminase and its mutants. Org Biomol Chem.

[CR13] Czarnievicz N, Rubanu MG, Iturralde M, Albarran-Velo J, Diamanti E, Gotor-Fernandez V, Skolimowski M, López-Gallego F (2022) A multiplex assay to assess the transaminase activity toward chemically diverse amine donors. ChemBioChem 202200614 10.1002/cbic.202200614 10.1002/cbic.202200614 36385460

[CR14] Daoud L, Ben Ali M (2020). Halophilic microorganisms: interesting group of extremophiles with important applications in biotechnology and environment. Inc.

[CR15] DasSarma S, DasSarma P (2015). Halophiles and their enzymes: negativity put to good use. Curr Opin Microbiol.

[CR16] Delgado-García M, Valdivia-Urdiales B, Aguilar-González CN, Contreras-Esquivel JC, Rodríguez-Herrera R (2012). Halophilic hydrolases as a new tool for the biotechnological industries. J Sci Food Agric.

[CR17] Dutta B, Bandopadhyay R (2022) Biotechnological potentials of halophilic microorganisms and their impact on mankind. Beni-Suef Univ J Basic Appl Sci 11 10.1186/s43088-022-00252-w 10.1186/s43088-022-00252-w PMC9152817 35669848

[CR18] Eslami M, Amoozegar MA, Asad S (2016). Isolation, cloning and characterization of an azoreductase from the halophilic bacterium *Halomonas elongata*. Int J Biol Macromol.

[CR19] Fang J, Zhang L, Bazylinski DA (2010). Deep-sea piezosphere and piezophiles: geomicrobiology and biogeochemistry. Trends Microbiol.

[CR20] Frillingos S, Linden A, Niehaus F, Vargas C, Nieto JJ, Ventosa A, Antranikian G, Drainas C (2000). Cloning and expression of α-amylase from the hyperthermophilic archaeon *Pyrococcus woesei* in the moderately halophilic bacterium *Halomonas elongata*. J Appl Microbiol.

[CR21] Gadda G, McAllister-Wilkins EE (2003). Cloning, expression, and purification of choline dehydrogenase from the moderate halophile *Halomonas elongata*. Appl Environ Microbiol.

[CR22] Galinski EA (1995) Osmoadaptation in bacteria. Adv Microb Physiol 272–328 10.1016/S0065-2911(08)60148-4 8540423

[CR23] Ghasemi A, Asad S, Kabiri M, Dabirmanesh B (2017). Cloning and characterization of *Halomonas elongata* L-asparaginase, a promising chemotherapeutic agent. Appl Microbiol Biotechnol.

[CR24] Heckmann CM, Dominguez B, Paradisi F (2021). Enantio-complementary continuous-flow synthesis of 2-aminobutane using covalently immobilized transaminases. ACS Sustain Chem Eng.

[CR25] Hegarty E, Paradisi F (2020). Implementation of biocatalysis in continuous flow for the synthesis of small cyclic amines. Chimia (aarau).

[CR26] Huffman MA, Fryszkowska A, Alvizo O, Borra-Garske M, Campos KR, Canada KA, Devine PN, Duan D, Forstater JH, Grosser ST, Halsey HM, Hughes GJ, Jo J, Joyce LA, Kolev JN, Liang J, Maloney KM, Mann BF, Marshall NM, McLaughlin M, Moore JC, Murphy GS, Nawrat CC, Nazor J, Novick S, Patel NR, Rodriguez-Granillo A, Robaire SA, Sherer EC, Truppo MD, Whittaker AM, Verma D, Xiao L, Xu Y, Yang H (2019). Design of an *in vitro* biocatalytic cascade for the manufacture of islatravir. Science (80-).

[CR27] Kastritis PL, Papandreou NC, Hamodrakas SJ (2007). Haloadaptation: insights from comparative modeling studies of halophilic archaeal DHFRs. Int J Biol Macromol.

[CR28] Katsimpouras C, Stephanopoulos G (2021). Enzymes in biotechnology: critical platform technologies for bioprocess development. Curr Opin Biotechnol.

[CR29] Kelefiotis-Stratidakis P, Tyrikos-Ergas T, Pavlidis IV (2019). The challenge of using isopropylamine as an amine donor in transaminase catalysed reactions. Org Biomol Chem.

[CR30] Kennedy SP, Ng WV, Salzberg SL, Hood L, DasSarma S (2001). Understanding the adaptation of *Halobacterium* species NRC-1 to its extreme environment through computational analysis of its genome sequence. Genome Res.

[CR31] Littlechild JA (2015). Enzymes from extreme environments and their industrial applications. Front Bioeng Biotechnol.

[CR32] Marchini V, Benítez‐Mateos AI, Hutter SL, Paradisi F (2022) Fusion of formate dehydrogenase and alanine dehydrogenase as an amino donor regenerating system coupled to transaminases. ChemBioChem 202200428 10.1002/cbic.202200428 10.1002/cbic.202200428 PMC9828552 36066500

[CR33] Martínez AT, Ruiz-Dueñas FJ, Camarero S, Serrano A, Linde D, Lund H, Vind J, Tovborg M, Herold-Majumdar OM, Hofrichter M, Liers C, Ullrich R, Scheibner K, Sannia G, Piscitelli A, Pezzella C, Sener ME, Kılıç S, van Berkel WJH, Guallar V, Lucas MF, Zuhse R, Ludwig R, Hollmann F, Fernández-Fueyo E, Record E, Faulds CB, Tortajada M, Winckelmann I, Rasmussen JA, Gelo-Pujic M, Gutiérrez A, del Río JC, Rencoret J, Alcalde M (2017). Oxidoreductases on their way to industrial biotransformations. Biotechnol Adv.

[CR34] Mesbah NM (2022). Industrial biotechnology based on enzymes from extreme environments. Front Bioeng Biotechnol.

[CR35] Mokashe N, Chaudhari B, Patil U (2018). Operative utility of salt-stable proteases of halophilic and halotolerant bacteria in the biotechnology sector. Int J Biol Macromol.

[CR36] Nakhaee N, Asad S, Khajeh K, Arab SS, Amoozegar MA (2018). Improving the thermal stability of azoreductase from *Halomonas elongata* by introducing a disulfide bond via site-directed mutagenesis. Biotechnol Appl Biochem.

[CR37] Ng HS, Wan P-K, Kondo A, Chang J-S, Lan JC-W (2023). Production and recovery of ectoine: a review of current state and future prospects. Processes.

[CR38] Ono H, Sawada K, Khunajakr N, Tao T, Yamamoto M, Hiramoto M, Shinmyo A, Takano M, Murooka Y (1999). Characterization of biosynthetic enzymes for ectoine as a compatible solute in a moderately halophilic eubacterium, *Halomonas elongata*. J Bacteriol.

[CR39] Oren A (2013). Life at high salt concentrations, intracellular KCl concentrations, and acidic proteomes. Front Microbiol.

[CR40] Pastor JM, Salvador M, Argandoña M, Bernal V, Reina-Bueno M, Csonka LN, Iborra JL, Vargas C, Nieto JJ, Cánovas M (2010). Ectoines in cell stress protection: uses and biotechnological production. Biotechnol Adv.

[CR41] Planchestainer M, Contente ML, Cassidy J, Molinari F, Tamborini L, Paradisi F (2017). Continuous flow biocatalysis: production and in-line purification of amines by immobilised transaminase from *Halomonas elongata*. Green Chem.

[CR42] Planchestainer M, Hegarty E, Heckmann CM, Gourlay LJ, Paradisi F (2019). Widely applicable background depletion step enables transaminase evolution through solid-phase screening. Chem Sci.

[CR43] Qvist J, Ortega G, Tadeo X, Millet O, Halle B (2012). Hydration dynamics of a halophilic protein in folded and unfolded states. J Phys Chem B.

[CR44] Research B (2021) BCC research report. Global markets for enymes in industrial applications. USA: BCC Research LLC. Bio030L

[CR45] Romero-Fernandez M, Heckmann CM, Paradisi F (2022). Biocatalytic production of a Nylon 6 precursor from caprolactone in continuous flow. Chemsuschem.

[CR46] Romero-Fernandez M, Paradisi F (2021). Biocatalytic access to betazole using a one-pot multienzymatic system in continuous flow. Green Chem.

[CR47] RouraPadrosa D, Benítez-Mateos AI, Calvey L, Paradisi F (2020). Cell-free biocatalytic syntheses of l -pipecolic acid: a dual strategy approach and process intensification in flow. Green Chem.

[CR48] RouraPadrosa D, Nissar Z, Paradisi F, Reactors DM (2021). Efficient amino donor recycling in amination reactions: development of a new alanine dehydrogenase in continuous flow and dialysis membrane reactors. Catalysts.

[CR49] RouraPadrosa D, De VV, Contente ML, Molinari F, Paradisi F (2019). Overcoming water insolubility in flow: enantioselective hydrolysis of Naproxen Ester. Catalysts.

[CR50] Saik RK, Gelfand DH, Stoffel S, Scharf SJ, Higuchi R, Horn GT, Mullis KB, Erlich HA (1987). Primer directed enzymatic amplification of DNA with a thermostable DNA polymerase. Science (80-).

[CR51] Sani RK, Rathinam NK (2018) Bioprospecting of extremophiles for biotechnology applications. In: Sani RK, Rathinam NK (eds) Extremophilic microbial processing of lignocellulosic feedstocks to biofuels, value-added products, and usable power. Springer International Publishing AG 1–308

[CR52] Sauer T, Galinski EA (1998) Bacterial milking: a novel bioprocess for production of compatible solutes. Biotechnol Bioeng 57:306–313 https:// doi/pdf/10.1002/(SICI)1097-0290(19980205)57:3<306::AID-BIT7>3.0.CO;2-L 10099207

[CR53] Tanimura K, Nakayama H, Tanaka T, Kondo A (2013). Ectoine production from lignocellulosic biomass-derived sugars by engineered *Halomonas elongata*. Bioresour Technol.

[CR54] Taran M, Rad M, Alavi M (2018) Biosynthesis of TiO2 and ZnO nanoparticles by *Halomonas elongata* IBRC-M 10214 in different conditions of medium. BioImpacts 8:81–89 10.15171/bi.2018.10 10.15171/bi.2018.10 PMC6026522 29977829

[CR55] Vreeland RH, Litchfield CD, Martin EL, Elliot E (1980). *Halomonas elongata*, a new genus and species of extremely salt-tolerant bacteria. Int J Syst Bacteriol.

[CR56] Wang D, Chen J, Wang Y, Du G, Kang Z (2021) Engineering *Escherichia coli* for high-yield production of ectoine. Green Chem Eng 0–6 10.1016/j.gce.2021.09.002

[CR57] Winkler CK, Schrittwieser JH, Kroutil W (2021). Power of biocatalysis for organic synthesis. ACS Cent Sci.

[CR58] Yin J, Chen JC, Wu Q, Chen GQ (2015). Halophiles, coming stars for industrial biotechnology. Biotechnol Adv.

[CR59] Yoo W, Kim B, Jeon S, Kim KK, Kim TD (2020). Identification, characterization, and immobilization of a novel YbfF esterase from *Halomonas elongata*. Int J Biol Macromol.

